# Carbohydrate utilization and metabolism is highly differentiated in *Agaricus bisporus*

**DOI:** 10.1186/1471-2164-14-663

**Published:** 2013-09-30

**Authors:** Aleksandrina Patyshakuliyeva, Edita Jurak, Annegret Kohler, Adam Baker, Evy Battaglia, Wouter de Bruijn, Kerry S Burton, Michael P Challen, Pedro M Coutinho, Daniel C Eastwood, Birgit S Gruben, Miia R Mäkelä, Francis Martin, Marina Nadal, Joost van den Brink, Ad Wiebenga, Miaomiao Zhou, Bernard Henrissat, Mirjam Kabel, Harry Gruppen, Ronald P de Vries

**Affiliations:** 1 CBS-KNAW Fungal Biodiversity Centre, Uppsalalaan 8, 3584 CT Utrecht, The Netherlands; 2 Wageningen University, Laboratory of Food Chemistry, Bomenweg 2, 6703 HD Wageningen, The Netherlands; 3 INRA, UMR1136 INRA/UHP, Interactions Arbres/ Micro-organismes, Centre de Nancy, Champenoux 54280, France; 4 University of Warwick, Warwick, CV35 9EF, Wellesbourne, UK; 5 Microbiology, Utrecht University, Padualaan 8, 3584 CH Utrecht, The Netherlands; 6 East Malling Research, New Road, East Malling, Kent ME19 6BJ, UK; 7 Wellcome Trust Centre for Human Genetics, University of Oxford, Roosevelt Drive, Oxford OX3 7BN, UK; 8 UMR 6098 CNRS–Universités Aix-Marseille I and II, Marseille Cedex 9 13288, France; 9 College of Science, University of Swansea, Singleton Park, Swansea SA2 8PP, UK; 10 Department of Food and Environmental Sciences, University of Helsinki, P. O. Box 56, 00014 Helsinki, Finland

## Abstract

**Background:**

*Agaricus bisporus* is commercially grown on compost, in which the available carbon sources consist mainly of plant-derived polysaccharides that are built out of various different constituent monosaccharides. The major constituent monosaccharides of these polysaccharides are glucose, xylose, and arabinose, while smaller amounts of galactose, glucuronic acid, rhamnose and mannose are also present.

**Results:**

In this study, genes encoding putative enzymes from carbon metabolism were identified and their expression was studied in different growth stages of *A. bisporus*. We correlated the expression of genes encoding plant and fungal polysaccharide modifying enzymes identified in the *A. bisporus* genome to the soluble carbohydrates and the composition of mycelium grown compost, casing layer and fruiting bodies.

**Conclusions:**

The compost grown vegetative mycelium of *A. bisporus* consumes a wide variety of monosaccharides. However, in fruiting bodies only hexose catabolism occurs, and no accumulation of other sugars was observed. This suggests that only hexoses or their conversion products are transported from the vegetative mycelium to the fruiting body, while the other sugars likely provide energy for growth and maintenance of the vegetative mycelium. Clear correlations were found between expression of the genes and composition of carbohydrates. Genes encoding plant cell wall polysaccharide degrading enzymes were mainly expressed in compost-grown mycelium, and largely absent in fruiting bodies. In contrast, genes encoding fungal cell wall polysaccharide modifying enzymes were expressed in both fruiting bodies and vegetative mycelium, but different gene sets were expressed in these samples.

## Background

Carbon catabolism serves fungi with energy in the form of reducing equivalents and ATP, as well as essential precursor metabolites for biosynthesis, such as glucose-6-phosphate and fructose-6-phosphate [1]. In nature plant biomass is the main carbon source for many fungal species. *A. bisporus* (the white button mushroom) is commercially cultivated on a composted mixture of lignocellulose-containing materials (mainly wheat straw and horse manure), which is highly selective for this fungus [2,3].

The major constituents of the lignocellulose fraction of compost are cellulose and the hemicellulose xylan (70% of the biomass) [4] and lignin [5-7]. Due to their diverse and complex polymeric nature, degradation of plant cell wall polysaccharides to their monomeric constituent requires a large range of enzymes [8,9]. Most of these enzymes have been divided into families in a classification system for Carbohydrate Active enZymes (CAZy, http://www.cazy.org) [10]. It has been shown that during mycelial growth and fruiting *A. bisporus* produces a range of extracellular enzymes, which are involved in the degradation of the lignocellulosic fraction in compost [11-14]. A shift in fungal metabolism takes place during development of the fruiting body of *A. bisporus* that is closely linked to an increased rate of cellulose and hemicellulose degradation [15]. The production of laccase and cellulase was suggested to be connected to the high rate and flow of carbon metabolism during fruiting body development [16,17]. Lignin degradation by *A. bisporus* decreases towards the end of the mushroom production cycle [18-20].

The major monosaccharide constituents of lignocellulose are D-glucose, D-xylose, and L-arabinose, while smaller amounts of D-galactose, D-galacturonic acid, L-rhamnose and D-mannose are also present. These monosaccharides are taken up by the fungal cell and converted through specific pathways [21]. Both L-arabinose and D-xylose catabolism are part of the pentose catabolic pathway [22], which ends at D-xylulose-5-phosphate, an intermediate of the pentose phosphate pathway (PPP). D-Glucose can enter several biochemical pathways [9,23,24], but can also lead to the synthesis of mannitol, trehalose and other storage compounds, such as glycogen and fatty acids [25]. The minor components of polysaccharides present in compost are converted through the galacturonic acid catabolic pathway [26], the D-galactose catabolic pathways (the Leloir pathway, the oxido-reductive pathway and the DeLey Doudoroff pathway) [27] and the L-rhamnose catabolic pathway [28].

Studies on carbon metabolism in *A. bisporus* have mainly focused on mannitol and trehalose. Synthesis of mannitol in *A. bisporus* is mediated by an NADPH-dependent mannitol dehydrogenase using fructose as substrate [29]. Metabolism trehalose involves either the trehalose synthase complex, [30], or trehalose phosphorylase (EC 2.4.1.64), which catalyze the reversible hydrolysis of trehalose into glucose-1-phosphate and glucose [30]. Remarkable differences were found in carbon metabolism of fruiting body and vegetative mycelium [31-34]. Mannitol functions as an osmolyte, which accumulates to high levels during fruiting body growth while after sporulation the level of mannitol decreases rapidly [35]. It might also serve as a post-harvest reserve carbohydrate [31,33,36]. Trehalose also serves as a reserve carbohydrate, which is present at lower levels than mannitol that decline during fruiting body development. It has been suggested that trehalose is synthesized in the mycelium and translocated to the fruiting body [16,32,34].

Gene expression analysis of genes encoding enzymes for polysaccharide modification and sugar metabolism offers an improved understanding of carbohydrate utilization and the metabolic fate of monosaccharides in the litter degrading fungus *A. bisporus*. Here, we identified genes encoding enzymes involved in carbon metabolism using the recently sequenced *A. bisporus* genome [37]. The expression of these genes and genes encoding plant biomass degrading enzymes was analyzed during different stages of growth of *A. bisporus*, revealing significant differences between mycelium grown on plates, in compost or in casing-soil, and fruiting bodies.

## Results

### Identification and expression analysis of genes encoding enzymes of central metabolism

The two sequenced genomes of *A. bisporus* var. *bisporus* H97 and var. *burnettii* JB137-s8 were analyzed to identify genes involved in central carbon metabolism. Identification was performed using the confirmed pathway genes from other fungi (Additional file 1).

Gene expression was assessed in mycelium grown on defined medium, in casing layer and in compost, and in fruiting bodies, using specific custom 60-mer Agilent microarrays (see "Methods"). Only those genes with > 2-fold differences and P-value <0.05 in gene expression between compost/casing layer/fruiting body and culture-grown mycelium were considered to be differentially expressed (Additional file 2).

#### ***Glycolysis & gluconeogenesis***

**Figure 1:**
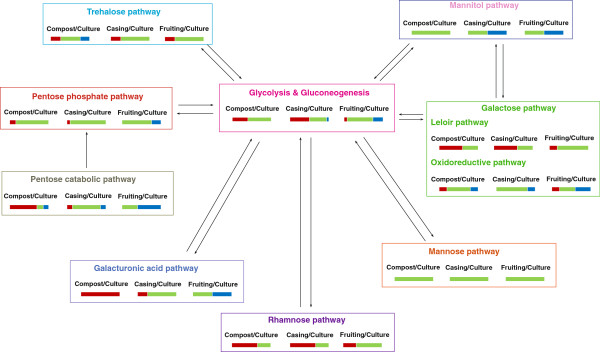
**Schematic representation of the expression of genes of the different carbon metabolic pathways.** Bars under the growth stages indicate the percentage of genes that are 2-fold upregulated (red), between 2-fold upregulated and 2-fold downregulated (green), and more than 2-fold downregulated (blue) in the sample compared to culture-grown mycelium.

Most genes from glycolysis were moderately upregulated in compost and casing compared to undifferentiated mycelium grown on agar medium, while their levels were similar or downregulated in the fruiting bodies (Figure 1, Additional files 3 and 4). In contrast, the gluconeogenic gene encoding phosphoenolpyruvate carboxykinase (PEPCK) was 8-fold upregulated in fruiting bodies.

#### ***Pentose phosphate pathway***

Expression of most PPP genes is similar in casing, compost and fruiting bodies compared to plate grown mycelium, while only some genes are slightly up- (in compost and casing layer) or down-regulated (in fruiting bodies) (Figure 1, Additional files 3 and 4). There is no consistent effect on either the oxidative or the non-oxidative part of the PPP.

#### ***Pentose catabolic pathway***

A significant increase in expression of most of the pentose catabolic pathway genes were detected in compost and to a lesser extent in the casing layer compared to plate grown mycelium, while their expression was reduced in fruiting bodies (Additional file 2). An exception was the putative L-xylulose reductase encoding gene that had reduced expression levels in compost and casing compared to plate-grown mycelium.

#### ***Catabolism of D-galactose, D-galacturonic acid, L-rhamnose and D-mannose***

**Figure 2:**
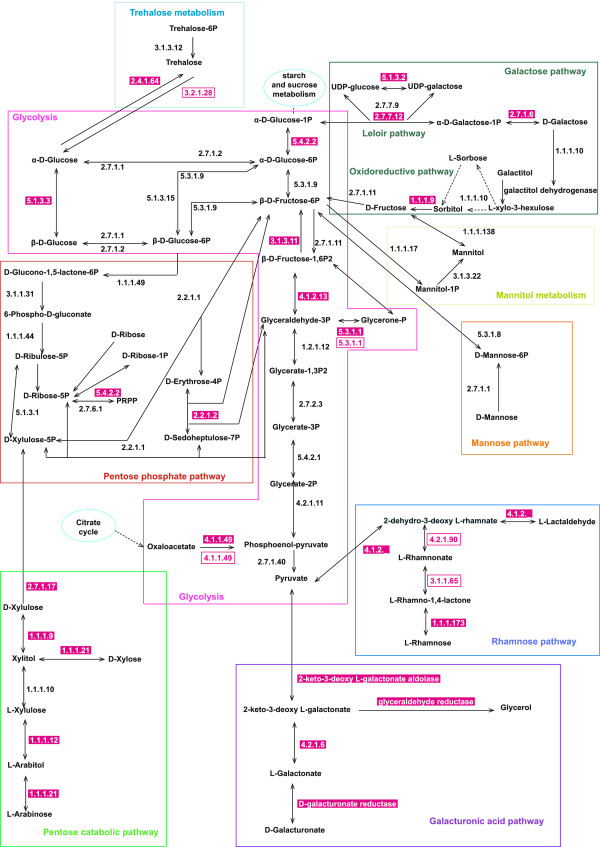
**Map of the central metabolism in *A. bisporus.*** Gene products contributing to these pathways are indicated. EC numbers in pink boxes indicate that genes encoding these enzymes are upregulated in compost (white numbers) or fruiting bodies (pink numbers) compared to plate-grown mycelium.

The putative *A. bisporus* genes of galacturonic acid catabolic pathway are strongly upregulated in compost and to a lesser extent in the casing layer, while they are down-regulated in fruiting bodies (Figure 2). Expression of genes from the D-galactose Leloir pathway was similar or elevated in all samples compared to plate-grown mycelium (Additional file 2). In contrast, nearly all genes of the D-galactose oxido-reductive pathway were upregulated in compost and downregulated in fruiting bodies (Additional file 2). Most genes from the rhamnose and mannose catabolic pathways (Additional file 1) [28] were similar or upregulated in compost, casing layer and fruiting bodies, compared to plate-grown mycelium (Additional file 2).

#### ***Mannitol and trehalose metabolism***

The mannitol-1-phosphate dehydrogenase encoding gene was similarly expressed in compost, casing layer and fruiting bodies, while the mannitol dehydrogenase encoding gene was similar in compost and downregulated in casing layer and fruiting body (Figure 2).

Expression of most trehalose metabolism genes was similar or upregulated in samples from compost and casing layer in comparison to undifferentiated plate-grown mycelium (Additional file 2). The exception was the gene encoding the neutral trehalase (EC 3.2.1.28), which was downregulated in compost. In samples from fruiting bodies, a gene encoding a neutral trehalase was slightly upregulated.

#### ***Organic acid metabolism***

Oxalic acid and citric acid are among the two most commonly produced organic acids by fungi [38]. No specific upregulation for oxalic acid metabolic genes was observed in any of the samples. In contrast, several of the citric acid metabolic genes were expressed at higher levels in fruiting bodies than in compost or the casing layer.

### Comparison of the expression of carbon metabolic genes between *A.bisporus* and *L. bicolor*

Orthologs of *A. bisporus* carbon metabolic genes were identified in the genome of a mycorrhiza species *L. bicolor* S238N (Additional file 1), with the exception of genes for L-rhamnose utilization genes for which no homologs could be found in *L. bicolor*.

The gene expression differentiation pattern of fruiting body versus mycelium was calculated for both fungi. In contrast to the prevalent gene downregulation in glycolysis, PPP and PCP pathways in *A. bisporus*, most of the genes in these pathways showed constant expression in mature fruiting bodies and free-living mycelium in *L. bicolor*.

### Expression of genes encoding plant cell wall polysaccharide degrading enzymes

Expression of genes encoding plant cell wall degrading enzymes from *A. bisporus* active against all the major plant cell wall polysaccharides was detected (Table 1). These genes are expressed at significantly higher levels in compost than in the other samples. For xylan and cellulose related genes, 90% and 64%, respectively, were expressed in compost while in casing layer and fruiting bodies less than 15% of these genes were expressed. In compost, expression of genes encoding enzymes targeting other polysaccharides (*e.g.* starch, pectin and xyloglucan) was also observed. Some genes of families GH5 and CE4, which contain enzymes acting on both plant and fungal cell wall polysaccharides, were upregulated in either compost or fruiting bodies.

**Table 1:** Percentage of plant degrading cell wall enzymes that are up regulated, number of genes expressed in compost, casing layer or fruiting bodies grouped by polysaccharide and their putative function

**Polysaccharide**	**CAZy families**	**No. genes**	**Compost**	**Casing layer**	**Fruiting bodies**
Xylan	GH10,11,43,115	19	89	5	5
	CE1,5,15				
Xyloglucan	GH12,21,31*,74,95	5	100	0	0
Cellulose	GH1*,5*,3,6,7,9,61	22	64	9	14
Chitin/xylan	CE4*	11	36	9	27
Pectin	GH2,28,35,51,53,78,88,105	26	96	12	4
	CE8,12				
	PL1,3,4				
Mannan	GH1*,5*,27	5	60	40	0
Starch	GH13,15,31*	15	31	0	19

*not all genes of the family are related to designated polysaccharide.

GH: Glycoside Hydrolase, CE: Carbohydrate Esterase, PL: Polysaccharide Lyase.

A detailed list of the genes of these CAZy can be found in Additional file 5.

### Expression of genes encoding fungal cell wall degrading/modifying enzymes

Fungal cell wall degrading and modifying enzymes have received less attention than plant cell wall degrading enzymes, resulting in a less well defined assignment of function. During growth *A. bisporus* needs to synthesize and modify its cell wall. As growth occurs in compost, casing layer and fruiting bodies, genes encoding fungal cell wall modifying enzymes need to be expressed in all growth stages. However, as the morphology of these stages is not identical, different genes may be expressed in compost and fruiting bodies. A complete list of genes encoding putative fungal cell wall modifying enzymes can be found in Additional file 5, including their putative function. Of all genes encoding putative fungal cell wall modifying enzymes 36% were expressed in all three samples, indicating a basal set of fungal cell wall modifying enzymes. Only 20% of the genes were upregulated in the compost, while about 30% were upregulated in the fruiting bodies. None of the genes were specifically upregulated in the casing layer.

Some CAZy families related to fungal cell wall modification contain genes that were upregulated in compost as well as genes that were upregulated in fruiting bodies. This applies in particular to GH16 (endo-1,3(4)-β-glucanase), GH17 (endo-1,3-β-glucosidase) and GH18 (chitinases). Genes specifically expressed in compost were found in GH5, GH55 and GH72. Most of the genes of GH92 (α-mannosidase) are upregulated in compost. Genes specifically expressed in fruiting bodies were found in GH63 (α-glucosidase) and GT17 (glucan endo-1,3-β-glucosidase). Most genes from GT2 (chitin synthase), GT48 (1,3-β-glucan synthase), GT57 (α-1,3-glucosyltransferase) and GT15 were also upregulated in fruiting bodies.

### Carbohydrate composition analysis of mycelium grown compost and casing layer and of fruiting bodies

Compost, casing layer and wheat straw were analysed for lignin, ash, protein, total carbohydrates and carbohydrate composition. Results are presented in Table 2. When the *A. bisporus* mushrooms have matured, compost consists of lignin (41% w/w) and ash (36% w/w), carbohydrates (17% w/w) and proteins (13%). Significant amounts of sandy particles and gravel are present in the compost and casing layer and due to the Klason lignin determination method we expect that some of this sandy inorganic material remained on the filter and is included in the calculated lignin amount [39]. The main monosaccharides released from compost by acid hydrolysis were xylose and glucose (4.4% w/w and 8.4% w/w, respectively). The composition of wheat straw was used as a reference for the composition of carbohydrates in raw compost as analysis showed that in raw compost the molar composition of carbohydrates is the same as in wheat straw (data not shown). The wheat straw composition determined in our study (Table 2) is in agreement with previously reported composition [40]. The molar composition of compost after mature mushrooms have been formed differs from that of wheat straw.

**Table 2:** Composition of wheat straw, compost and casing layer

**%w/w (based on dry matter)**	**Wheat straw**	**Compost**	**Casing layer**
Lignin (Klason)	27	41 ^a^	52^a^
Total carbohydrates	57	17	12
Ash	5	36	29
Protein (%N *6.25)	3	13	7
**Carbohydrate composition (molar%)**			
Arabinose	6.0	5.6	1.6
Xylose	42.6	30	14
Mannose	0.89	4	6.1
Galactose	1.34	3.3	7
Rhamnose	0.8	1.4	2
Glucose	45	47	60
Uronic acids	3.9	8.6	9.2
Acetic acid (mol Ac/100 mol Xyl)	32	12	9

^a^Small part of inorganic material is included.

The casing layer is a mixture of calcium and peat that consists mainly of lignin (52% w/w) and ash (29% w/w). There are few carbohydrates present (14% w/w) and the main monosaccharides released after acid hydrolysis were xylose (1.4% w/w), mannose (0.6% w/w) and glucose (7.5% w/w) [41]. As mentioned above, the actual lignin amount is likely to be lower than measured due to calcium and sandy particles that remain on the filter after acid hydrolysis.

Aqueous extraction of compost, casing layer and fruiting bodies revealed that more than 95% of carbohydrates are insoluble. A high performance anion exchange (HPAEC) elution pattern of water extract from mycelium grown compost, casing layer and fruiting bodies was used to analyse the extract.

Changes in free soluble monosaccharides were observed in these samples. Concentrations of arabinose, galactose and xylose were high in compost, while only traces of these monosaccharides were found in casing layer and fruiting bodies (Table 3). High levels of glucose were observed in all samples. Mannitol and trehalose levels were significantly higher in fruiting bodies than in compost and casing layer (Table 3), as were the levels of citric acid (data not shown), while no oxalic acid was detected in the samples. The very high level of sorbitol in the compost samples could suggest a role as a transportable carbon compound from the vegetative mycelium to the fruiting body (Table 3).

**Table 3:** Concentration (mg/kg) of free (soluble) monosaccharides, trehalose, mannitol and sorbitol

**Component (mg/kg fresh material)**	**Compost**	**Casing layer**	**Fruiting body**
Arabinose	37.4	3.0	3.5
Rhamnose	7. 9	1.5	1.1
Galactose	15.9	6.1	2.3
Glucose	819.9	224.3	149.4
Xylose	221.9	11.6	5.0
Mannose	23.8	10.2	7.9
Fructose	70.4	41.7	703
Sorbitol	7654	3160	5242
Mannitol	3994	1657	20298
Trehalose	397	140	1064

**Figure 3:**
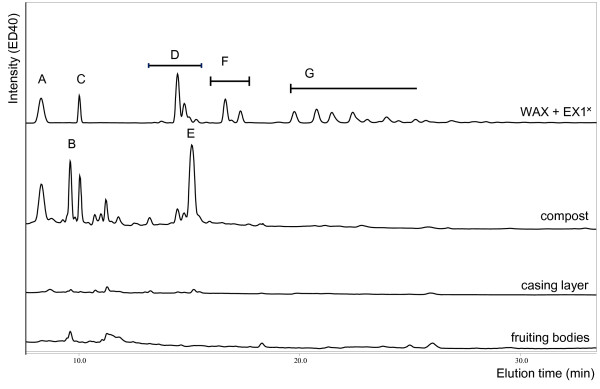
**HPAEC elution patterns of the water soluble fraction of compost, casing layer and fruiting bodies (xylobiose (A), cellobiose (B), xylotriose (C), single substituted xylo-oligomers (D), likely xylo-oligomers with GlcA substituent (E), double substituted xylo-oligomers (F), multiple substituted xylo-oligomers (G).** Water extract of fruiting body was diluted 20 times. *WAX + EX1- digest of wheat arabinoxylan and endoxylanase I [42].

Soluble oligosaccharides were detected in the compost, while none were detected in the casing layer or fruiting bodies (Figure 3). The peaks detected in the compost were compared to standards of xylo- and cello-dextran oligosaccharides (DP 2–6) and the elution pattern of the well described endoxylanase I digest of wheat arabinoxylan in order to identify them [42]. Mainly xylobiose (Figure 3 B), xylotriose (Figure 3 D), and presumably xylo-oligomers with attached glucuronic acid or its 4-O-methyl ether (Figure 3 F) were found. In addition to xylo-oligomers, cellobiose was detected. The small peaks that were detected are likely xylo- and cello-oligomers of higher degree of polymerisation and arabinose substituted xylo-oligomers.

## Discussion

In this study, genes encoding carbon metabolic genes were identified in the genome of *A. bisporus* and their expression in different growth stages was compared to the available carbohydrates and the expression of genes encoding carbohydrate modifying enzymes.

### Compost is mainly focused on degrading plant biomass

Analysis of the expression of genes encoding plant and fungal polysaccharide modifying enzymes identified in the *A. bisporus* genome [37] revealed correlation between these genes and composition of carbohydrates. Expression analysis of CAZy-genes demonstrated that in compost the highest expressed genes are related to (hemi-) cellulose and pectin degradation, while also some genes related to β-1,3-glucan modification were expressed. A large decrease of carbohydrate content and, therefore, polysaccharides was revealed in the compost after growth of *A. bisporus* and fruiting body production. Expression data supports that the decrease in carbohydrates observed is partially caused by the growth of *A. bisporus*. About 90% of the genes encoding xylan degrading enzymes were upregulated in the compost. This correlates well with the detection of soluble xylo-oligosaccharides in compost. Higher proportions of arabinose and xylose in the water extracts of compost than in the water extracts of casing layer and fruiting bodies (Table 3) are in good agreement with the expression of genes encoding arabinofuranosidases, endoxylanases and β-xylosidases. The presence of xylo-oligomers in compost suggests that the β-xylosidase activity may be the limiting factor in xylose release. The pentose catabolic pathway was strongly upregulated in the compost and moderately upregulated in the casing layer, while it was downregulated in the fruiting bodies. This confirms the relevance of release and conversion of these pentoses as a main carbon source for *A. bisporus* during growth in compost.

Expression of genes encoding other plant polysaccharide degrading enzymes that are not normally associated with compost, e.g. starch, pectin and xyloglucan related genes, was also detected. In nature *A. bisporus* can grow on various substrates ranging from leaf litter and soil under cypress in coastal California to manured soil, composts of plant debris, and other horticultural and agricultural situations reported in Europe [43]. Growth on these different substrates is likely due to the ability of *A. bisporus* to produce a wide range of plant polysaccharide degrading enzymes and it may co-express genes aimed at different polysaccharides. Such a system is well described for the ascomycete *Aspergillus niger*, in which a single regulator (XlnR) activates the expression of genes related to cellulose, xylan and xyloglucan degradation [44,45]. For this fungus six regulators involved in plant polysaccharide degradation have been described and they usually respond to the presence of the monomeric building blocks of the polysaccharides [44,46-50]. While no homologs of these regulators have been found in basidiomycetes (Todd and de Vries, unpublished data), it is likely that basidiomycetes have developed similar systems using different regulators.

### The casing layer serves as an intermediate phase

In the casing layer, which is a mixture of peat and lime, it is likely that the detected glucose and mannose at least partially drive from the mycelial cell wall, in the form of glucans and mannoproteins, respectively. While some genes encoding putative plant cell wall degrading enzymes were expressed in the casing layer, the level of up-regulation compared to plate-grown mycelium is much smaller than that in compost. In addition, expression of some chitinase encoding genes was detected. The casing layer seems to be an intermediate phase in which some genes related to plant biomass degradation are expressed, but also modification of the *A. bisporus* cell wall is an important process for the conversion to fruiting body morphology. The lack of soluble polysaccharides indicates that the role of the mycelium in the casing layer is mainly to supply carbohydrates to the fruiting body.

### The fruiting body focuses on modification of fungal polysaccharides

For *A. bisporus* growth and development a basal set of fungal cell wall modifying enzymes is needed and about 36% of the genes encoding such enzymes were expressed in mycelium grown compost, casing layer and fruiting bodies. The other expressed genes encoding fungal cell wall modifying enzymes are upregulated during specific growth stages. This suggests that *A. bisporus* has specific genes for mycelium development and growth and others for fruiting body formation and modification. Some genes from GH16 (encoding endo-1,3(4)-β-glucanase), GH17 (encoding glucan endo-1,3-β-glucosidase) and GH18 (encoding chitinases) are upregulated in the compost while others from the same families are upregulated in the fruiting bodies. These results support the compositional and morphological differences found between mycelium and fruiting bodies [35]. Expression of different sets of genes encoding fungal cell wall modifying enzymes has also been described for other fungi. For example, in *A. niger* different sets of genes encoding chitinases, chitin synthases and alpha-1.3-glucan synthases were expressed in the centre and the periphery of plate grown cultures [51].

Enzymes from families GH5 and CE4 have several described activities, some of which are related to plant cell wall polysaccharides, while others are related to fungal cell wall polysaccharides (http://www.cazy.org). For some of the enzymes from these families upregulation in compost was observed, while others were upregulated in fruiting bodies. A strong correlation was observed between the putative function and the expression of genes from these families. While genes encoding putative plant biomass degrading enzymes were upregulated in compost, genes encoding putative fungal cell wall modifying enzymes were upregulated in fruiting bodies (Additional file 6).

### Carbon metabolism is partially differentiated in *A. bisporus*

Expression analysis demonstrated that the pentose catabolic pathway and galacturonic acid pathway were strongly upregulated in compost and moderately upregulated in the casing layer, while they were downregulated in fruiting bodies. Most genes of the oxido-reductive galactose pathway were also higher expressed in compost than in fruiting bodies, which correlates with a higher galactose level in compost compared to fruiting bodies. In contrast to the pathways described above, the glycolytic pathway and PPP are active in all growth stages of *A. bisporus*. This correlates well with the presence of free glucose in all samples, suggesting that hexose catabolism is an important factor in all growth stages of *A. bisporus*. The PPP has been described as the major route of glucose catabolism in fruiting bodies of *A. bisporus*[35,52,53] as well as *Lentinula edodes*[54] as a greater proportion of glucose oxidation occurs via the PPP in the fruiting body than in vegetative mycelium, while glycolysis has been suggested to be the major pathway of sugar metabolism during fruiting body development in *Pleurotus ostreatus, Coprinus cinereus* and *Schizophyllum commune*[55-57].

The concentration of mannitol in fruiting bodies was six times higher than in compost. However, expression of mannitol pathway genes was significantly lower in fruiting bodies than in compost, suggesting that mannitol is synthesized in the vegetative mycelium and transported to the fruiting body. Earlier studies observed that mannitol functions as an osmoregulatory compound and facilitates a continuous influx of water from compost to the fruiting body to support turgor and fruiting body development [58,59]. This would suggest that mannitol is unlikely to be transported by diffusion from the mycelium. Therefore, it should either be transferred by active transport or alternatively, be synthesized in the fruiting body. If the latter is the case, a possible explanation for the observed expression of the genes could be that the encoded enzymes are transported into the fruiting body.

Trehalase activity was reported to be highest during the peak of each flush, while low activity was detected during the interflush period [16], which correlates well with the highest expression of a putative trehalase encoding gene in fruiting bodies of our study. In contrast, trehalose phosphorylase was found to increase during the interflush period [34], which was also confirmed by the expression analysis in our study.

No significant differences were observed in the expression of genes related to oxalic acid metabolism in the different growth stages and the expression levels suggest that oxalic acid formation occurs in all stages. The high expression of one of the putative oxalate decarboxylase encoding genes could explain why no oxalic acid was detected in the samples as this could imply that degradation of oxalic acid occurs at least as fast as its synthesis. It should also be noted that only free oxalic acid was analysed in this study, while oxalic acid present in the form of calcium oxalate was not included.

In contrast, several of the genes involved in citric acid metabolism are higher expressed in the fruiting body than in compost and casing layer, which correlates well with the higher levels of citric acid that were detected in these samples. As citric acid is known to have preservative properties against bacteria in food [60], it is tempting to speculate that the accumulation of citric acid in fruiting bodies may also be involved in the defence mechanism of the mushroom against bacteria. Another explanation may be the high respiration rates of the fruiting bodies, which requires high expression of genes associated with the citric acid/Krebs cycle and mitochondria in general [54]. High expression of isocitrate lyase was also reported in brown-rot fungi, where this enzyme produced succinate and glyoxylate from isocitrate [61,62]. Progressive downregulation of this gene was observed in the casing layer during the shift from vegetative mycelium to fruiting body [63].

### The difference in carbon metabolism between *A. bisporus* and *L. bicolor*

Comparison of two basidiomycetes *A. bisporus* and *L. bicolor* didn’t show any correlation in expression of carbon metabolic genes. This could be explained by the difference in life styles of these two species. As a saprobe, *A. bisporus* is highly dependent on obtaining carbon from its surroundings. In contrast, the mycorrhizae *L. bicolor* obtains carbon from its symbiotic partner in the form of sucrose, placing a much lower demand on a versatile carbon metabolism.

## Conclusions

The data from our study demonstrates that overall there is a clear correlation between expression of genes related to plant and fungal polysaccharides and the ability of *A. bisporus* to degrade these polysaccharides. We see a clear difference in genes expressed within mycelium grown compost and fruiting bodies supporting the hypothesis that different genes are expressed in *A. bisporus* mycelium and fruiting bodies. This supports previous results that this fungus produces different enzymes during its life cycle [64]. However, it should also be recognised that gene expression is likely to be dynamic and here we have examined it at the time point when first flush was harvested (approximately 34 days after compost was inoculated with spawn). Large oscillations of cellulase activity in the compost have been observed which co-ordinate with mushroom fruiting body production and oscillations of activities of fruiting body metabolic enzymes [16,17,65].

Moreover, our study demonstrates a clear correlation between the expression of genes encoding plant and fungal cell wall polysaccharides with the composition of carbohydrates in compost, casing layer and fruiting bodies. Genes encoding plant cell wall polysaccharide degrading enzymes were mainly expressed in compost-grown mycelium, and largely absent in fruiting bodies. In contrast, genes encoding fungal cell wall polysaccharide modifying enzymes were expressed in both fruiting bodies and vegetative mycelium in the compost, but different gene sets were expressed in these samples.

In the present study an *in silico* metabolic reconstruction of the central carbon metabolism in *A. bisporus* was performed and combined with expression analysis of the relevant genes in different growth stages of *A. bisporus*. The analysis of metabolic pathways in *A. bisporus* may provide information about the requirements of carbon source and energy metabolism during commercial growth of *A. bisporus*. We showed that during growth in compost and casing a much larger variety of carbon sources was used by *A. bisporus* than during growth on synthetic medium. In contrast, carbon metabolism in fruiting bodies appears to be mainly aimed at hexoses. This could indicate that only these sugars are transported towards the fruiting body from the vegetative mycelium, which implies that carbon transport to the fruiting bodies is a highly regulated and selective process.

## Methods

### Materials used

Compost, casing layer and fruiting bodies cultures were harvested at the first flush stage of *A. bisporus* strain A15 and were stored at -20°C. Samples (about 100 g) were collected, freeze dried and milled (<1 mm) (Retsch Mill MM 2000, Retsch, Haan, Germany). Duplicates were mixed in ratio 1:1. Wheat straw was collected as raw material and a representative sample was made by mixing 16 different freeze dried and milled samples of wheat straw in the same ratio. All chemicals, unless stated otherwise were obtained from Sigma, Merck or Fluka (Busch, Switzerland).

### Water extraction

Milled compost, casing layer and fruiting bodies (0.4 g) were suspended in millipore water (20 mL) and boiled at 100°C for 10 min to inactivate enzyme activity, shaken vigorously and filtered (0.2 μm). The filtrate was used to analyse water soluble carbohydrates.

### Analytical and spectrometric methods

#### ***Neutral carbohydrate composition***

Neutral carbohydrate composition of wheat straw, compost and casing layer was analysed according to Englyst [66] using inositol as an internal standard. Samples were treated with 72% (w/w) H_2_SO_4_ (1h, 30°C) followed by hydrolysis with 1M H_2_SO_4_ for 3h at 100°C and the constituent sugars released were derivatised and analysed as their alditol acetates using gas chromatography (GC). The amount of neutral carbohydrates was corrected for mannitol, sorbitol and trehalose.

#### ***Uronic acid content***

Uronic acids content of wheat straw, compost and casing layer was determined as anhydro-uronic acid by an automated m-hydroxydiphenyl assay [67] using an autoanalyser (Skalar Analytical BV, Breda, The Netherlands). Glucuronic acid was used as a reference.

#### ***Lignin content***

Samples of wheat straw, compost and casing layer were analysed for acid insoluble (Klason) lignin. To each sample of 300 mg (dry matter) 3 ml of 72% (w/w) H_2_SO_4_ was added and samples were pre-hydrolysed for 1 h at 30°C. After this pre-hydrolysis, 37 ml of distilled water was added and samples were put in a boiling water bath for 3 h and shaken every half hour. Further, suspension was filtered over G4 glass filters (Duran Group GmbH, Mainz, Germany). The residual part was washed until it was free of acid and dried overnight at 105°C. The weight of the dried residual part was taken as a measure of the acid insoluble lignin content.

#### ***Protein content***

Nitrogen content of wheat straw, compost and casing layer was analysed using the combustion (DUMAS) method on a Flash EA 1112 Nitrogen Analyser (Thermo Scientific, Rockford, IL, USA). Methionine (Acros Organics, New Jersey, USA) was used as a standard and protein content was calculated from the nitrogen content of the material, using a protein conversion factor of 6.25 [68].

#### ***Ash content***

Samples of wheat straw, compost and casing layer (0.5 g) were dried in the oven overnight (105°C), weighed and put in the oven on 504°C overnight. Next day samples were weighed and difference between the mass at 105°C and 504°C was taken as ash content.

### Chromatographic methods

#### ***Analysis of soluble carbohydrates, sorbitol, trehalose and mannitol***

High-performance anion-exchange chromatography (HPAEC) was performed on an Ultimate 3000 system (Dionex, Sunnyvale, CA, USA) equipped with a CarboPac PA-1 column (2 mm x 250 mm ID) in combination with a CarboPac guard column (2 mm x 50 mm ID) and PAD detection. System was controlled by the Chromelion software (Dionex).

Separation and quantification of monosaccharides was done at a flow rate 0.4 ml/min, and the mobile phase consisted of (A) 0.1 M NaOH, (B) 1 M NaOAc in 0.1 M NaOH and (C) H_2_O. The elution profile was as follows: 0–40 min 100% C; 40.1-45.1 min from 100% A to 100% B, 45.1-50 min 100% B, 50.1-58 min 100% A, 58.1-73 min 100% C. From 0 to 40 min and from 58 to 73 min post column addition of 0.5 M NaOH at 0.1 ml/min was performed to detect and quantify the eluted saccharides.

Soluble carbohydrates. sorbitol, mannitol and trehalose were separated on the same system, including columns and detection. The flow rate used to separate sorbitol, mannitol and trehalose was 0.3 mL/min, and the mobile phase consisted of (A) 0.1 M NaOH, (B) 1 M NaOAc in 0.1 M NaOH and (C) H_2_O. The elution profile was as follows: 0–5 min 100% A, 5-25% 0-30% B, 25.1-30 min 100% B, 30–50 min 100% A.

Water soluble oligosaccharides were separated with a combination of linear gradients from two types of eluents, A: 0.1 M NaOH and B: 1 M NaOAc in 0.1 M NaOH. The elution profile was as following: 0–35 min: 0-38% B, cleaning step 3 min 100% B and equilibration step 12 min 100% A. As a reference for xylo-oligomers with substitution, elution pattern of wheat arabinoxylan (medium viscosity, Megazyme, Bray, Ireland) digest with a pure and well described endoxylanase I was used [42,69], while as a standard for cellulose and xylan oligomers, cellodextrans and xylodextrans were used. Water extract of compost and casing layer were injected on the column without dilution and fruiting body water extract was diluted 20 times before injecting it on the column.

#### ***Organic acid analysis***

Oxalic acid and citric acid were determined with an Ultimate system (Dionex, Sunnyvale, USA) equipped with a Shodex RI detector and an Aminex HPX 87H column (300 mm x 7.8 mm) (Bio-Rad, Hercules, CA, USA) plus pre-column [70]. Elution was performed by using 5 mM H_2_SO_4_ as eluent at a flow rate of 0.6 ml min^-1^ at 40°C.

#### ***Esterified acetic acid content***

Samples of compost and casing layer (20 mg) were saponified with 1 mL of 0.4 M NaOH in isopropanol/H_2_0 (1:1) for 3 h at room temperature. The acetic acid content was determined with an Ultimate system (Dionex) equipped with a Shodex RI detector and an Aminex HPX 87H column (300 mm x 7.8 mm) (Bio-Rad) plus pre-column [70]. Elution was performed by using 5 mM H_2_SO_4_ as eluent at a flow rate of 0.6 mL min^-1^ at 40°C. The level of acetic acid substituents was corrected for the free acetic acid in the sample.

### Genome annotation and comparative genomics

*A. bisporus* var *bisporus* (http://genome.jgi.doe.gov/Agabi_varbisH97_2/Agabi_varbisH97_2.home.html), *A. bisporus* var *burnetti* (http://genome.jgi.doe.gov/Agabi_varbur_1/Agabi_varbur_1.home.html), *Aspergillus niger*, *Aspergillus oryzae*, *Aspergillus nidulans*, *Phanerochaete chrysosporium* or *Postia placenta* and *Laccaria bicolor* S238N genomes (http://genome.jgi-psf.org/Lacbi2/Lacbi2.home.html) were used to perform genomic comparisons. Full genome clusters of orthologous genes were created by OrthoMCL (http://www.ncbi.nlm.nih.gov/pubmed/12952885) with E-value 1e-5 and sequence matching coverage 60% as the cutoff (http://www.ncbi.nlm.nih.gov/pubmed/20152020). Carbon catabolic genes of *Agaricus* and *Laccaria* were identified by extracting the orthologous clusters containing known carbon catabolic genes from *Aspergulli*, *P. chrysosporium* or *P. placenta*.

### Transcriptome analysis

Gene expression was profiled in the commercial (heterokaryon) strain A15. *A. bisporus* strain A15 was grown in compost made from wheat straw, chicken litter and gypsum in the proportions 10:6:0.5 w/w. The first phase of composting was with regular mixing and took approximately 25 days. At phase II of composting process compost was pasteurized with steam at 70°C for 7 days. Phase II compost was inoculated with 1–2% w/w *A. bisporus* mycelium spawn, placed in 50 kg growth trays, and incubated at 25°C, 95% relative humidity for 21 days. The colonised compost was covered by 5 cm peat-based casing layer and incubated for a further 7 days. The culture samples refer to axenic culture and the media used was compost extract medium [71]. Fresh pasteurised compost was oven dried for 48 h at 80°C. Dried compost was boiled in distilled water (7.5 g / l) for 1 h and cooled to room temperature. After centrifugation (5000 rpm, 20 min), the supernatant was used to make the medium [72]. Peptone (0.5% w/v) was added to the extract and the medium buffered to pH 7 using potassium phosphate buffer.

The fruiting body samples represent the mature mushroom stage 2 with a stretched, unbroken veil fruiting body (including the stipe, cap and *pilei pellis* (skin) tissues) [35]. The casing samples consisted of a mixture of mycelium aggregates, undifferentiated primordia (1–2 mm circular with no differentiation between stipe and cap tissues), differentiated primordia (~ 7 mm diameter, oval with some evidence of cap tissue differentiation). The compost samples represent the mycelium growing in wheat straw compost. The samples for RNA extraction were collected on separate occasions from separate mushroom houses. Four biological replicates of each developmental stage were analyzed [37].

RNA was prepared from fruiting body and culture samples using a standard Trizol protocol. RNA was extracted from compost and casing samples using a method based on the FastRNA Pro Soil-Direct kit (MP Biochemicals) [63]. RNA was quantified using a NanoDrop-1000 spectrophotometer and quality was monitored with the Agilent 2100 Bioanalyzer (Agilent Technologies, Santa Clara, CA).

Custom arrays (Agilent ID 027120) were developed using 10,438 CDS (filtered model set) from the H97 v2 gene annotation; 5 x 60-mer oligos per CDS and the 8 x 60K randomised format were designed using the Agilent eArray software. Cyanine-3 (Cy3) labeled cRNA was prepared from 0.6 ug RNA using the Quick Amp Labelling kit (Agilent) according to the manufacturer’s instructions, followed by RNAeasy column purification (QIAGEN, Valencia, CA). Dye incorporation and cRNA yield were checked with the NanoDrop ND-1000 Spectrophotometer. 600 ng of Cy3-labelled cRNA (specific activity >10.0 pmol Cy3/ug cRNA) was fragmented at 60°C for 30 minutes in a reaction volume of 25 μl containing 1x Agilent fragmentation buffer and 2x Agilent blocking agent following the manufacturer’s instructions. On completion of the fragmentation reaction, 25 μl of 2x Agilent hybridization buffer was added to the fragmentation mixture and hybridized to Agilent arrays (ID 027120) for 17 hours at 65°C in a rotating Agilent hybridization oven. After hybridization, microarrays were washed 1 minute at room temperature with GE Wash Buffer 1 (Agilent) and 1 minute with 37°C GE Wash buffer 2 (Agilent) then 10 seconds in acetonitrile and 30 seconds in Stabilization and drying solution (Agilent). Slides were scanned immediately after washing on the Agilent’s High-Resolution C Scanner (G2505C US94100321) using one color scan setting for 8 x 60K array slides (Scan resolution 3um). The scanned images were analyzed with Feature Extraction Software (Agilent) using default parameters (protocol GE1_107_Sep09 and Grid: 027120_D_F_20100129) to obtain background subtracted and spatially detrended Processed Signal intensities. Features flagged in Feature Extraction as Feature Non-uniform outliers were excluded [37]. Only those genes with > 2-fold differences and P-value <0.05 in gene expression between compost/casing layer/fruiting body and culture-grown mycelium were considered to be differentially expressed. Comparison of ratios of compost/culture transcript profiles was used to identify the most highly upregulated transcripts found in mycelium grown on compost during vegetative growth. The comparison of compost/fruiting body transcript profiles highlights developmental stage differences during mushroom formation [37].

The *Laccaria bicolor* S238N transcriptomes of 2 weeks free-living mycelium (FLM) and mature fruiting bodies were extracted from Gene Expression Omnibus (GEO) by series number GSE9784. Gene expression profiles were extracted, normalized and analysed as described previously [73]. Only genes with 2-fold differences and P-value <0.05 were considered significantly differentially expressed.

### Availability of supporting data

Micro array data from *Agaricus bisporus* and *Laccaria bicolor* used in this paper is available at GEO, accession number GSE39569 (http://www.ncbi.nlm.nih.gov/geo/query/acc.cgi?acc=GSE39569) and GSE32559 (http://www.ncbi.nlm.nih.gov/geo/query/acc.cgi?acc=GSE32559), respectively.

## Competing interests

The authors declare that they have no competing interests.

## Authors’ contributions

AP and EJ analysed the data and drafted the manuscript. EJ, WdB, HG and MK performed and analysed carbohydrate compositions experiments. AB, KSB, MPC, DCE, AK and FM performed transcriptome analysis. PMC and BH performed comparative genomic analysis of CAZy genes. MZ performed the genomic and transcriptomic comparisons between *A. bisporus* and *L. bicolor*. AP, EB, BSG, MRM, MN, JvdB and AW analysed the *A. bisporus* genome for metabolic pathways. RPdV designed the study. All authors participated in data interpretation, and read and approved the final manuscript.

## Supplementary Material

Additional file 1 **Genes encoding putative enzymes of carbon metabolism in *A. bisporus *and *L. bicolor.*** Genes in *A. bisporus* var. *bisporus* were identified using orthologous clustering method based on best bi-derectional hits of all-vs-all blast to the genomes included in analysis. Click here for file

Additional file 2 **Expression comparison of carbon metabolic genes in different growth stages of *A. bisporus *var. *bisporus *and *L. bicolor*.** In the ratio between the values genes that are upregulated compared to culture-grown mycelium are in pink, while genes that are down regulated are in green. Click here for file

Additional file 3 **Proportion of upregulated genes of the different carbon metabolic pathways in compost, casing layer and fruiting bodies.** Venn diagrams represent different carbon metabolic pathways indicating the percentage of genes that are 2-fold upregulated in the samples compared to culture-grown mycelium. Click here for file

Additional file 4 **Proportion of downregulated genes of the different carbon metabolic pathways in compost, casing layer and fruiting bodies.** Venn diagrams represent different carbon metabolic pathways indicating the percentage of genes that are 2-fold downregulated in the samples compared to culture-grown mycelium. Click here for file

Additional file 5 **Expression of genes encoding putative fungal and plant polysaccharide modifying enzymes.** Putative functions are based on CAZy family assignment and homology to characterised enzymes. The activity on plant of fungal polysaccharides is putative and not always supported biochemically. The expression levels are the average of 4 biological replicates. Click here for file

Additional file 6 **Maximum likelihood tree showing the correlation between plant biomass degrading and fungal cell wall modifying enzymes and upregulation of genes encoding these enzymes in compost or fruiting body.** Phylogenetic tree of the members of CE4 (A) and GH5 (B) families together with characterized enzymes was based on maximum likelihood method with 1000 bootstraps replications and WAG substitution model. Text in pink boxes shows that genes encoding indicated enzymes are upregulated in compost/fruiting body. Click here for file
