# Supplementary material for: Carbohydrate utilization and metabolism is highly differentiated in Agaricus bisporus
Source: BMC Genomics. 2013 Sep 30;14:663. doi: 10.1186/1471-2164-14-663 (PMC3852267; doi:10.1186/1471-2164-14-663)

Additional file 3. Proportion of upregulated genes of the different carbon metabolic pathways in compost, casing layer and fruiting bodies.
Venn diagrams represent different carbon metabolisc pathways indicating the percentage of genes that are 2-fold upregulated in the samples compared to culture-grown mycelium.

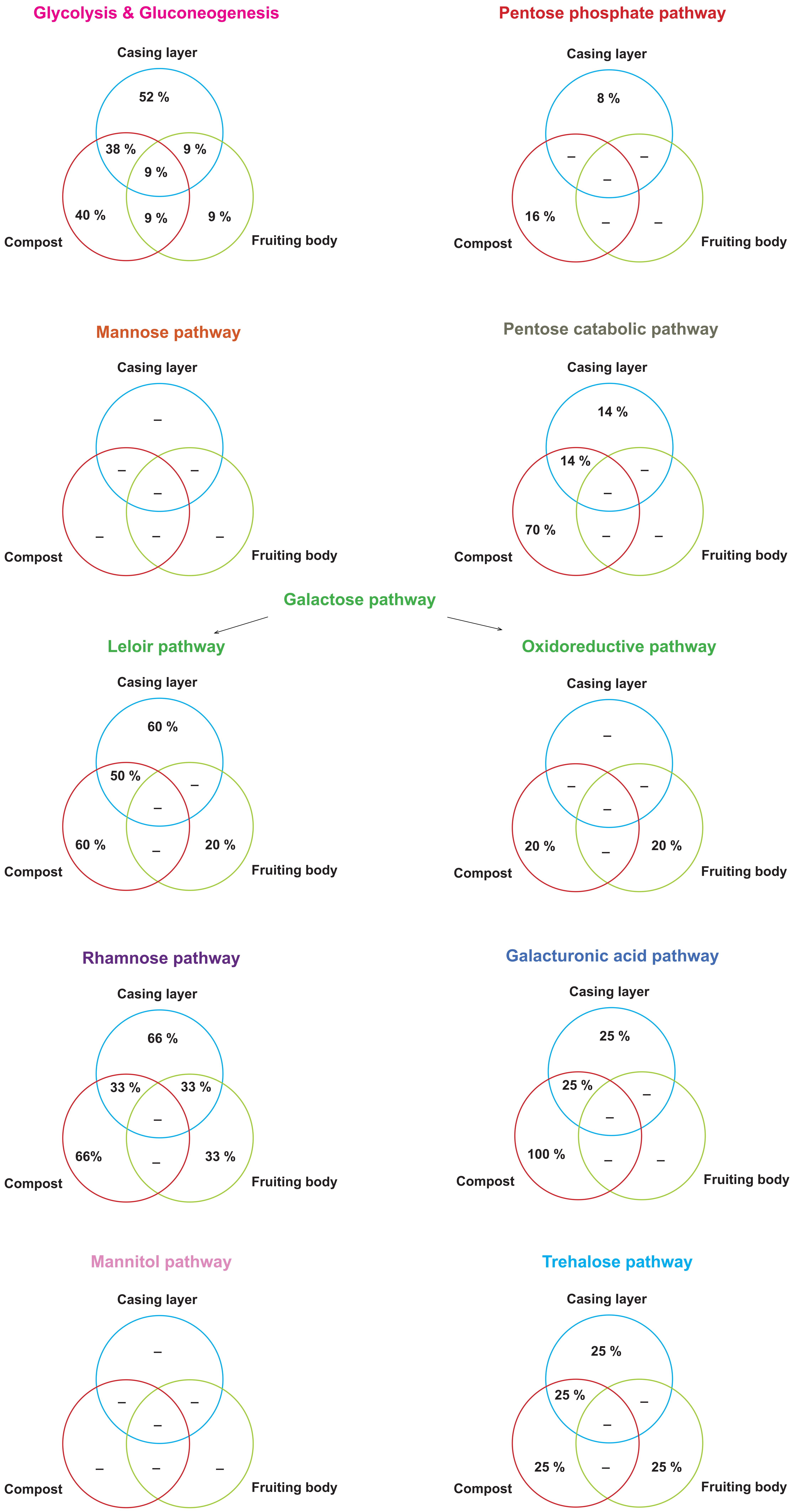

Supplement: Additional file 3 — Proportion of upregulated genes of the different carbon metabolic pathways in compost, casing layer and fruiting bodies. Venn diagrams represent different carbon metabolic pathways indicating the percentage of genes that are 2-fold upregulated in the samples compared to culture-grown mycelium. [file 1471-2164-14-663-S3.pdf]
