# Supplementary material for: Carbohydrate utilization and metabolism is highly differentiated in Agaricus bisporus
Source: BMC Genomics. 2013 Sep 30;14:663. doi: 10.1186/1471-2164-14-663 (PMC3852267; doi:10.1186/1471-2164-14-663)

Additional file 6. Maximum likelihood tree showing the correlation between plant biomass degrading and fungal cell wall modifying enzymes and upregulation of genes encoding these enzymes in compost or fruting body.
Phylogenetic tree of the members of CE4 (A) and GH5 (B) families together with characterized enzymes was based on maximum likelihood method with 1000 bootstraps replications and WAG substitution model.
Text in pink boxes shows that genes encoding indicated enzymes are upregulated in compost/fruiting body.

A

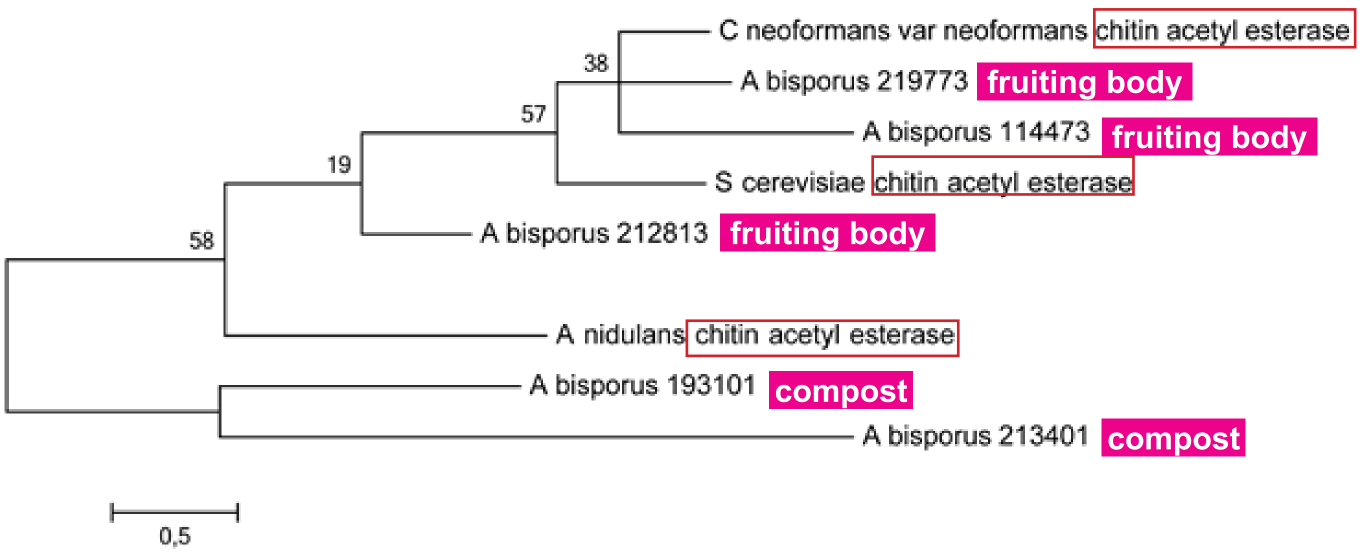

B

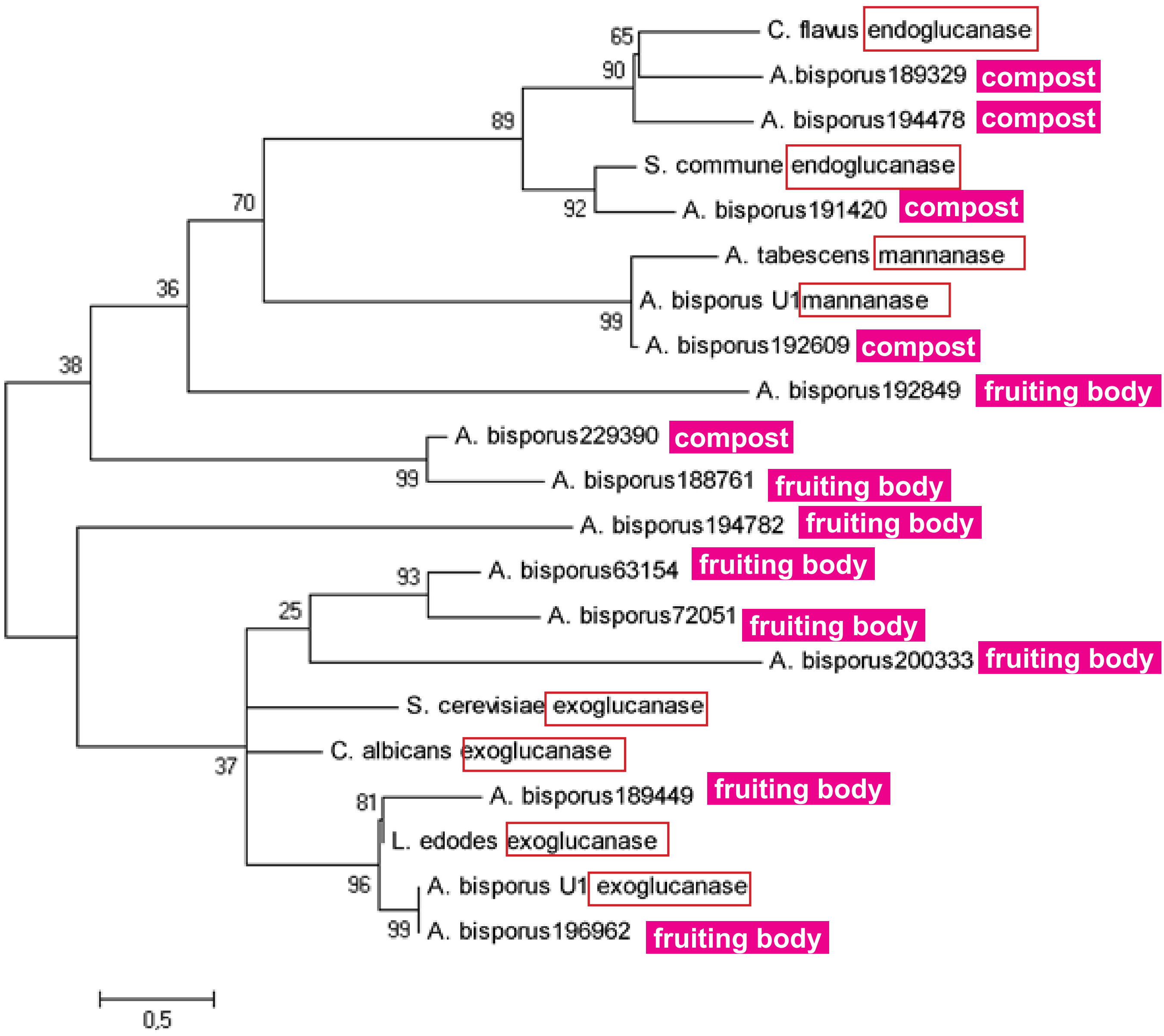

Supplement: Additional file 6 — Maximum likelihood tree showing the correlation between plant biomass degrading and fungal cell wall modifying enzymes and upregulation of genes encoding these enzymes in compost or fruiting body. Phylogenetic tree of the members of CE4 (A) and GH5 (B) families together with characterized enzymes was based on maximum likelihood method with 1000 bootstraps replications and WAG substitution model. Text in pink boxes shows that genes encoding indicated enzymes are upregulated in compost/fruiting body. [file 1471-2164-14-663-S6.pdf]
